# Developmental regulation of spatio-temporal patterns of cortical circuit activation

**DOI:** 10.3389/fncel.2012.00065

**Published:** 2013-01-04

**Authors:** Trevor C. Griffen, Lang Wang, Alfredo Fontanini, Arianna Maffei

**Affiliations:** ^1^Program in Neuroscience, Stony Brook UniversityStony Brook, NY, USA; ^2^Medical Scientist Training Program, Stony Brook UniversityStony Brook, NY, USA; ^3^Department of Neurobiology and Behavior, Stony Brook UniversityStony Brook, NY, USA; ^4^SUNY Eye Research ConsortiumNY, USA

**Keywords:** visual cortex, microcircuitry, synaptic plasticity, GABA, NMDA, AMPA, postnatal development, signal propagation

## Abstract

Neural circuits are refined in an experience-dependent manner during early postnatal development. How development modulates the spatio-temporal propagation of activity through cortical circuits is poorly understood. Here we use voltage-sensitive dye imaging (VSD) to show that there are significant changes in the spatio-temporal patterns of intracortical signals in primary visual cortex (V1) from postnatal day 13 (P13), eye opening, to P28, the peak of the critical period for rodent visual cortical plasticity. Upon direct stimulation of layer 4 (L4), activity spreads to L2/3 and to L5 at all ages. However, while from eye opening to the peak of the critical period, the amplitude and persistence of the voltage signal decrease, peak activation is reached more quickly and the interlaminar gain increases with age. The lateral spread of activation within layers remains unchanged throughout the time window under analysis. These developmental changes in spatio-temporal patterns of intracortical circuit activation are mediated by differences in the contributions of excitatory and inhibitory synaptic components. Our results demonstrate that after eye opening the circuit in V1 is refined through a progression of changes that shape the spatio-temporal patterns of circuit activation. Signals become more efficiently propagated across layers through developmentally regulated changes in interlaminar gain.

## Introduction

During postnatal development, neural circuits in sensory cortices are extensively refined in an experience-dependent fashion (Carmignoto and Vicini, 1992; Katz and Shatz, 1996; Finnerty et al., 1999; Morales et al., 2002; Hensch, 2004; Espinosa and Stryker, 2012). The onset of sensory experience is known to promote the maturation of sensory circuits. In rodent primary visual cortex (V1), the time between eye opening and the fourth postnatal week is characterized by changes in receptor expression (Carmignoto and Vicini, 1992; Nase et al., 1999; Corlew et al., 2007; Yashiro and Philpot, 2008), gene expression (Lyckman et al., 2008), neurotransmitter release (Morales et al., 2002), connectivity (Blue and Parnavelas, 1983), mechanisms for plasticity (Ramoa and Sur, 1996; Huang et al., 1999; Rozas et al., 2001; Wang et al., 2012), and cell intrinsic properties (Etherington and Williams, 2011; Lazarus and Huang, 2011; Wang et al., 2012). The developmental regulation of synaptic and intrinsic properties in different cell types contributes to establishing mature connectivity and is thought to be the underpinning mechanism for the acquisition of mature visual processing (Hensch, 2004; Li et al., 2012). Specifically, the acquisition of a balance between excitation and inhibition is considered crucial to healthy circuit wiring (Hensch and Fagiolini, 2005). Shifts in the excitatory/inhibitory balance may affect not only the excitability of single neurons, but also how stimuli are propagated in the cortical circuit (Rozas et al., 2001; Wang et al., 2011).

Previous findings from a number of cortical regions have shown that synaptic components that, in V1, are strongly affected by early postnatal experience, such as synaptic transmission mediated by GABA and NMDA receptors, can significantly affect the spatio-temporal patterns of signal propagation (Laaris et al., 2000; Sato et al., 2008). These studies suggest the importance of excitatory and inhibitory synaptic transmission for the spread of circuit activation in neocortex, but do not assess whether these patterns of activation are modulated during development. In auditory cortex, passive tone rearing was shown to modify tonotopic maps of cortical activation in an experience-dependent fashion (Barkat et al., 2011). In addition, during postnatal development the dynamics of V1 responsiveness to repeated white matter stimulation are altered, and the experience-dependent maturation of cortical inhibition is believed to underlie this change (Rozas et al., 2001). Consistent with this we have shown that in V1 the spatio-temporal patterns of circuit activation are extensively modulated by changes in visual drive during the critical period, and depend on changes in the cortical level of inhibition (Wang et al., 2011). These data suggest that changes in synaptic properties during development may contribute to sculpting the patterns of activation of the circuit. How spatio-temporal patterns of visual cortical activation change during postnatal development under conditions of normal rearing is unknown.

Here we use voltage-sensitive dye (VSD) imaging in acute rat brain slices containing V1 to determine how the spatio-temporal propagation of activity within and between cortical layers is modulated during early postnatal development. VSD imaging provides high spatial and temporal resolution of cortical circuit activity, allowing us to quantify patterns of intracortical circuit activation in response to electrical stimulation (Laaris et al., 2000; Tominaga et al., 2000; Petersen and Sakmann, 2001; Sato et al., 2008; Barkat et al., 2011; Wang et al., 2011; Wester and Contreras, 2012). To bypass thalamocortical projections, which undergo significant postnatal refinement (Kato et al., 1983; Carmignoto and Vicini, 1992; Antonini and Stryker, 1993), and measure the spatio-temporal patterns of intracortical signal propagation, we directly stimulate L4 and measure the voltage changes in V1. We show that the spatio-temporal propagation of stimuli is significantly affected by the maturation of the circuit. During the time window between eye opening and the peak of the critical period for visual cortical plasticity (P13–P28) the activation of V1 is characterized by a progressive decrease in the peak and duration of the optical voltage signal that is mediated by changes in synaptic transmission. As the circuit in V1 matures, the signal leaving L4 becomes amplified upon reaching L2/3, suggesting an increase in the gain of the vertical signal propagation. Our results indicate that the maturation of healthy cortical circuits occurs through a series of events leading to temporally and spatially refined propagation of signals across layers. These developmentally regulated changes in circuit activation likely contribute to a progressive acquisition of mature visual processing (Fagiolini et al., 1994; Huang et al., 1999; Prévost et al., 2010; Li et al., 2012).

## Materials and methods

### Animals and acute slice preparation

All experimental procedures were approved by the Stony Brook University Animal Use Committee and followed National Institute of Health guidelines. Long–Evans rats obtained from Charles River, aged P13–P28 were used for recordings and were grouped as follows: P14 ± 2, P20 ± 1, and P27 ± 1. Acute slices containing V1 were prepared (Maffei et al., 2004, 2006; Maffei and Turrigiano, 2008; Wang et al., 2011, 2012). Slices were mounted on Omnipore filters and placed in a container filled with artificial cerebrospinal fluid (ACSF) continually perfused with a gas mixture of 95% CO_2_ and 5% O_2_ to maintain oxygenation and humidity (Tominaga et al., 2000; Wang et al., 2011). Slices were maintained at 37°C for approximately 20 min and then at room temperature.

### Voltage-sensitive dye staining and imaging

VSD staining and imaging was performed as previously described (Tominaga et al., 2000; Wang et al., 2011). Di-4-ANEPPS (Invitrogen; absorption: 496 nm, emission: 705 nm) dissolved in a 2:1 mixture of ethanol and 10% Cremophor-EL solution (v/v in ddH_2_O, Sigma) was prepared as stock solution (final concentration of 3.3 mg/ml) and stored at 4°C for no longer than 3 months. On the day of recording, a small volume of the stock solution was dissolved in a 1:1 mixture of fetal bovine serum (Sigma) and oxygenated ACSF (final di-4-ANEPPS concentration: 0.2 mM). Prior to recording, the slice was covered with 100 μl of VSD solution and allowed to incubate for 40 min at room temperature. The excess dye was washed off and the slice was placed in a 1 ml recording chamber mounted on an upright microscope (Olympus, BX51WI) and held in place with a flattened platinum ring.

For recording, a constant flow of oxygenated, 35°C ACSF was perfused into the chamber at a rate of 1.5 ml/min and the slice was allowed to sit for 10 min to wash out excess dye prior to the start of recording (Wang et al., 2011). A halogen lamp (150 W, TH 4-100, Olympus) with an electronically controlled shutter (Smart Shutter with Lambda 10B controller, Sutter Instruments) was used to activate the dye and detect voltage signals. The excitation light was first passed through an excitation filter (λ530 ± 10 nm), then projected onto a dichroic mirror (λ = 565 nm) and finally projected through the objective lens to illuminate the slice. The fluorescent signal generated by the tissue was passed through an absorption filter (λ = 590 nm) to a CCD camera connected to a PC via an I/O interface (MiCAM 02, SciMedia, Brainvision). A high numerical aperture 4× objective (NA 0.28, Olympus), reduced with a 0.5× lens on the c-mount of the CCD camera, was used to visually identify the region for imaging. The image resolution was 60 × 88 active pixels, with single pixel size of 20 μm, for a total imaged area of 1.2 × 1.8 mm. For each stimulus, 256 frames were acquired at 400 Hz, for a total of 640 ms. Stimuli were repeated 16 times with 15 s intervals, and the signal was averaged across the 16 repetitions. A peristaltic pump (Watson-Marlow Sci 400) was used to maintain a constant volume of ACSF in the recording chamber and prevent changes in the depth of focus. The shutter controller and the stimulus isolation unit were driven by the I/O interface of the CCD camera and the duration of the stimuli was controlled though the MiCam data acquisition software (Brainvision).

Extracellular stimulation was delivered with a tungsten unipolar electrode covered with a glass pipette (0.1 MΩ, Harvard Apparatus) and inserted below the surface of the slice to allow for stable recordings. The electrode was positioned at the center of the region identified as V1 in the lower portion of L4, as visually identified by laminar distinctions. The reference electrode was in the bath. 0.2 ms unimodal pulses were delivered at a stimulation intensity of 50 μA, which allowed for comparison of VSD signals between treatment groups (Wang et al., 2011).

### Analysis and statistics

Analysis of the VSD signal was performed with procedures developed in Image J, SigmaPlot (Systat Software, Inc.) and Igor Pro (WaveMetrics). To allow for comparisons between different conditions, voltage signals were measured as the transition in fluorescence from the baseline (ΔF/F), and signals were normalized to the initial background measured over the 25 ms immediately preceding each stimulus. VSD signals measured as ΔF/F have been shown to correlate with voltage changes measured intracellularly and with local field potentials (Laaris et al., 2000; Tominaga et al., 2000; Petersen and Sakmann, 2001; Wang et al., 2011).

**Figure 1:**
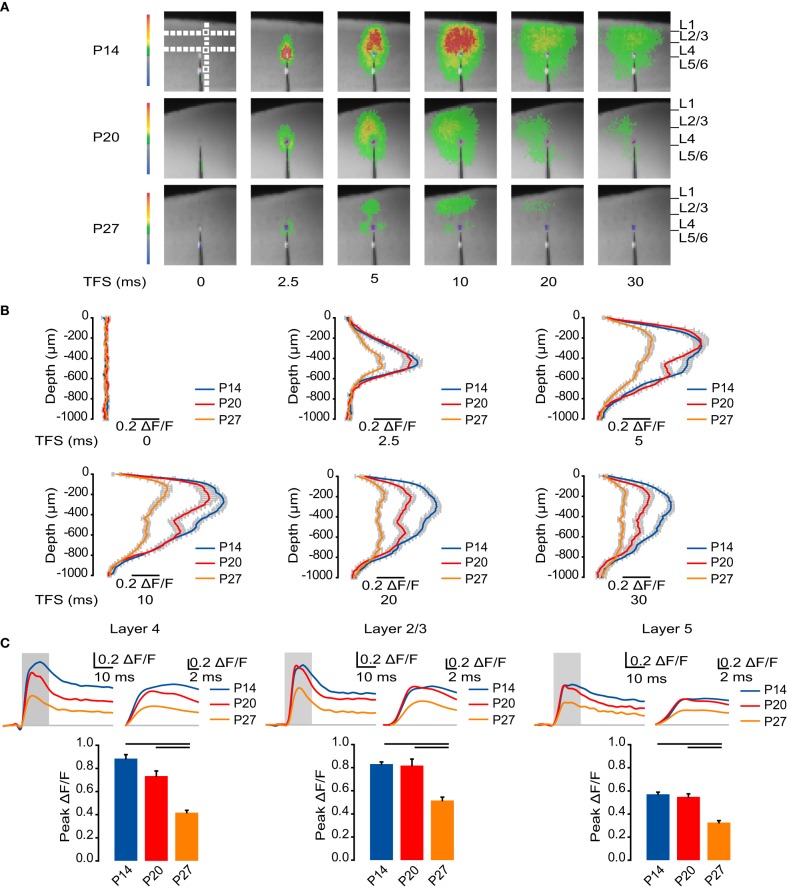
**Developmental reduction in cortical activation. (A)** Representative sample VSD images at 0, 2.5, 5, 10, 20, and 30 ms from L4 stimulation for each age group. Images were cropped to better visualize the activated region (from 60 × 88 to 45 × 50 pixels, 20 μm per pixel). Top left panel: White boxes: ROIs quantified in panel **(C)**, Figures 2, 3, 5, 6, and 7. Vertical white dashed line: ROI quantified in panel **(B)**. Horizontal white dashed lines: ROIs quantified in Figure 4. **(B)** Time course of the ΔF/F measured by line scans perpendicular to the pial surface. Blue: P14. Red: P20. Orange: P27. Error bars: ± SEM. **(C)** Top: Time course of optical signals measured from ROIs in L4, L2/3, and L5 from 10 ms before stimulation to 50 ms after stimulation on the left. The gray box indicates TFS 0 to 15 ms, which is shown amplified in the traces on the right to highlight changes in the time to peak. Blue: P14. Red: P20. Orange: P27. Light gray line: 0.0 ΔF/F. Bottom: Peak ΔF/F measured from ROIs in L4, L2/3, and L5. Blue: P14. Red: P20. Orange: P27. Error bars: ± SEM. Dark bars indicate significant changes, *p* < 0.05.

Interlaminar spread of the VSD signal was analyzed with line scans 3 pixels wide (60 μm) from the pial surface to a depth of 1000 μm (50 pixels) positioned next to the stimulating electrode and perpendicular to the pia (Figure 1A). Regions of interest (ROIs) 2 × 2 pixels (40 × 40 μm) were selected to analyze the time course of activation in L4, L2/3, and L5 over 50 ms as follows: the L2/3 ROI was placed at the point of maximal activation in L2/3. The L4 ROI was placed as close to the stimulating electrode as possible while avoiding the stimulation artifact from the electrode. The L5 ROI was placed 600 μm below the pial surface (Figure 1A). All three ROIs were aligned vertically, perpendicular to the pial surface. Threshold for signal detection was set at ±2 standard deviations from the baseline. Signals not reaching threshold were set to 0 ΔF/F for analysis.

Horizontal spread of the VSD optical signal was analyzed by fitting line scans 60 μm wide through L2/3, at the peak of horizontal spread, and L4, above the stimulating electrode (Figure 1A), with the following one-dimensional Gaussian equation using Igor Pro (WaveMetrics):

$$Y\left(x\right)\;=a\text{ exp}-\left[{\left(x-b\right)}^{\text{2}}/c^{\text{2}}\right]$$

where *a* is the amplitude of the curve, *b* is the offset of the peak, and *c* is the width (Wang et al., 2011).

Data are presented as mean ± SEM. *N* = 13 slices from 4 rats for the P14 group, *n* = 10 slices from 3 rats for the P20 group, except for the drug application experiments, where *n* = 9, and *n* = 11 slices from 3 rats for the P27 group. Statistical analysis was performed using SigmaPlot (Systat Software, Inc.). To determine significance, the Kruskal–Wallis One-Way ANOVA on Ranks was performed, and if significant, was followed by Dunn's test. *P*-values < 0.05 were considered significant.

### Solutions and drugs

ACSF contained (mM): NaCl 126, KCl 3, MgSO_4_ 2, NaHPO_4_ 1, NaHCO_3_ 25, CaCl_2_ 2, and dextrose 25. The pH was adjusted to 7.2 by bubbling with a gas mixture of 95% CO_2_ and 5% O_2_. To dissect the synaptic receptor components of the signal, the slices were then perfused cumulatively with blockers of NMDA, AMPA, and GABA_A_ receptors (Wang et al., 2011). The following drugs were delivered in additive sequence in ACSF (μM): APV 50 (Tocris), DNQX 20 (Tocris), and picrotoxin 20 (Tocris).

## Results

We studied the effects of development on the spatio-temporal propagation of activity through the cortical circuit. We used optical imaging of VSD coupled with electrical stimulation in L4 of V1 acute slice preparations from three age groups: P14, P20, and P27.

### Reduced circuit activation across layers during development

To begin investigating the effects of neurodevelopment during normal rearing on the spatio-temporal pattern of circuit activation, we first examined how activity elicited by direct stimulation of L4 spreads to other cortical layers in each age group (Figure 1A). Across all ages, circuit activation was centered on the site of stimulation in L4 at time from stimulus (TFS) 2.5 ms (Figure 1B). By TFS 5 ms the signal spread beyond L4 and peaked in L2/3. Activation of L5 also became apparent by TFS 5 ms (Figure 1B). Following the fast activating component of the optical signal, there was a persisting component with slow decay that became apparent after the fast component had died out by TFS 30 ms in all layers (Figure 1C).

To quantitatively assess circuit activation, we measured the time course of the VSD signal intensity for ROIs in L4, L2/3, and L5 (Figure 1C). In L4 at P14 the time to peak was 8.7 ± 0.5 ms, significantly slower than in the later developmental windows (Figure 1C; P20: 6.0 ± 0.6 ms; P27: 6.1 ± 0.5 ms; Dunn's test: P14 vs. P20, *p* < 0.05; P14 vs. P27, *p* < 0.05). As development progressed, the amplitude of the peak of L4 activation was significantly reduced, from a ΔF/F of 0.89 ± 0.034 at P14 and 0.73 ± 0.044 at P20 to 0.42 ± 0.020 at P27 (Figure 1C; Dunn's test: P14 vs. P27, *p* < 0.05; P20 vs. P27, *p* < 0.05).

From L4, activity propagated to L2/3. At P14, the time to peak of the optical signal in L2/3 was 10.2 ± 0.3 ms, significantly slower than 7.0 ± 0.3 ms at P20 and 8.2 ± 0.4 ms at P27 (Figure 1C; Dunn's test: P14 vs. P20, *p* < 0.05; P14 vs. P27, *p* < 0.05). In L2/3, the peak of the VSD signal was not significantly different between P14 and P20; however, by P27 there was a significant reduction in the peak amplitude (Figure 1C; P14: 0.83 ± 0.019; P20: 0.82 ± 0.056; P27: 0.52 ± 0.029; Dunn's test: P14 vs. P27, *p* < 0.05; P20 vs. P27, *p* < 0.05).

Activity induced by L4 stimulation propagated to deep layers at all ages. The time to peak in L5 was 8.8 ± 0.6 ms at P14, 7.5 ± 0.8 ms at P20, and 8.0 ± 0.7 ms at P27, indicating that there was no significant difference in the time to peak activation of deep layers between age groups (Figure 1C; ANOVA on ranks: *p* = 0.34). As in L4 and L2/3, peak activation in L5 did not change significantly from P14 to P20, but was significantly reduced by P27 (Figure 1C; P14: 0.57 ± 0.019; P20: 0.55 ± 0.026; P27: 0.33 ± 0.017; Dunn's test: P14 vs. P27, *p* < 0.05; P20 vs. P27, *p* < 0.05).

These data show that from eye opening through the fourth postnatal week, the magnitude of activation of the cortical circuit is reduced. As development progresses, the circuit becomes less strongly activated; however, temporal propagation of the signal becomes faster as the peak activation is reached more quickly in L4 and L2/3 in the older age groups.

### Signal persistence decreases across development

**Figure 2:**
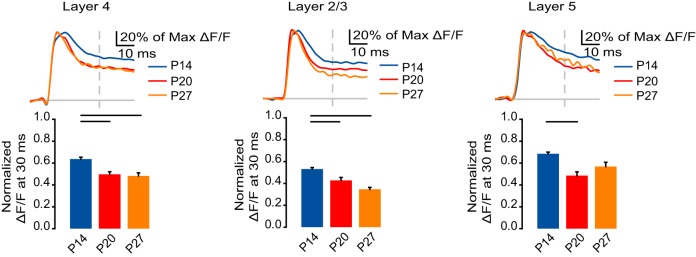
**Developmental reduction in signal persistence. Top:** Time course of optical signals from ROIs in L4, L2/3, and L5 from 10 ms before stimulation to 50 ms after stimulation normalized to the peak ΔF/F measured in each ROI. Blue: P14. Red: P20. Orange: P27. Light gray line: 0.0 ΔF/F. Light gray dash: TFS 30 ms. **Bottom:** ΔF/F at 30 ms normalized to the peak ΔF/F measured from ROIs in L4, L2/3, and L5. Blue: P14. Red: P20. Orange: P27. Error bars: ± SEM. Dark bars indicate significant changes, *p* < 0.05.

In all layers and at all ages, the VSD signal has fast and slow components, corresponding to the fast peak of activation followed by a slow phase of signal persistence. We have previously shown that this slow phase is present in both local field potential and VSD recordings in our preparation and can be almost entirely eliminated by blocking AMPA and NMDA receptors (Wang et al., 2011). To determine the time course of cortical activation and quantify possible developmental differences in the persistence of the voltage signal within each layer following stimulation, we calculated the ratio of the VSD signal at each time point to the peak signal (Figure 2). We compared the fractions of the peak signal that remained at 30 ms, a time point after the fast component of the VSD response has died out.

Within L4, a larger portion of the signal persisted at 30 ms at P14 compared to P20 and P27 (Figure 2; P14: 0.64 ± 0.017; P20: 0.50 ± 0.024; P27: 0.48 ± 0.029; Dunn's test: P14 vs. P20, *p* < 0.05; P14 vs. P27, *p* < 0.05). Likewise, in L2/3, the VSD signal persisted most in the P14 group, and significantly less in the P20 and P27 groups (Figure 2; P14: 0.53 ± 0.013; P20: 0.43 ± 0.028; P27: 0.35 ± 0.017; Dunn's test: P14 vs. P20, *p* < 0.05; P14 vs. P27, *p* < 0.05). In L5, there was a significant decrease in the persistence of the signal from P14 to P20 and a trend toward a reduction at P27 (Figure 2; P14: 0.69 ± 0.015; P20: 0.49 ± 0.035; P27: 0.57 ± 0.039; Dunn's test: P14 vs. P20, *p* < 0.05). While we did not directly compare the signal persistence between layers, the slope of the slow phase in L2/3 appears less steep than in L4 or L5, which might be a result of the relatively greater intralaminar connectivity within L2/3 (Burkhalter, 1989). These data show that as the animal gets older the activation of V1 becomes temporally restricted, suggesting a more time constrained propagation of activity in the cortical circuit.

### Interlaminar signal gain increases during development

**Figure 3:**
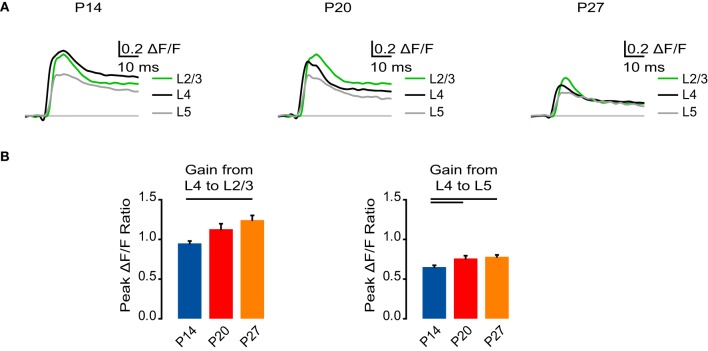
**Developmental increase in interlaminar gain. (A)** Time course of optical signals measured from ROIs at P14, P20, and P27 from 10 ms before stimulation to 50 ms after stimulation. Green: L2/3. Black: L4. Gray: L5. Light gray line: 0.0 ΔF/F. The unique color scheme of this panel reflects data organized by cortical layer, whereas in other panels, the color scheme reflects data organized by developmental age group. **(B)** Gain from L4 to L2/3 and L5 measured as the ratio of the peak ΔF/F from a ROI in L2/3 or L5 to the peak ΔF/F in a ROI in L4. Blue: P14. Red: P20. Orange: P27. Error bars: ± SEM. Dark bars indicate significant changes, *p* < 0.05.

The decrease in intensity of the VSD signal in the later stages of development that we examined could imply L4 stimulation is not as effective at activating V1. To address this, we examined the ratio of VSD signals from ROIs in L2/3 and L5 to ROIs in L4 in each age group as a measure of signal gain (Figure 3). From P14 to P27, signal gain from L4 to L2/3 increased significantly (Figure 3B; P14: 0.95 ± 0.029; P20: 1.13 ± 0.069; P27: 1.24 ± 0.059; Dunn's test: P14 vs. P27, *p* < 0.05). The signal gain from L4 to L5 was also increased in the older age groups (P20 and P27) compared to P14 (Figure 3B; P14: 0.63 ± 0.022; P20: 0.76 ± 0.035; P27: 0.78 ± 0.024; Dunn's test: P14 vs. P20, *p* < 0.05; P14 vs. P27, *p* < 0.05). These results suggest that while L4 stimulation elicited a smaller activation of all layers in older animals, feedforward activation actually became amplified. Thus, during development the cortical circuit acquires the capacity to propagate a temporally more restricted signal with increased gain.

### Intralaminar horizontal circuit activation is unchanged throughout development

**Figure 4:**
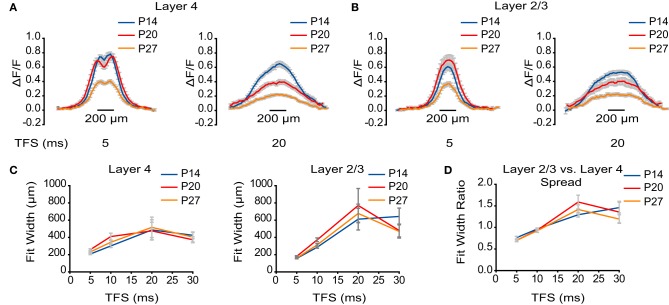
**No developmental change in horizontal spread of VSD signal. (A)** ΔF/F measured by line scans through L4 at TFS 5 and 20 ms. Blue: P14. Red: P20. Orange: P27. Error bars: ± SEM. **(B)** ΔF/F measured by line scans through L2/3 at TFS 5 and 20 ms. Blue: P14. Red: P20. Orange: P27. Error bars: ± SEM. **(C)** Plot of the width of activation in L4 and L2/3 at different TFS. Blue: P14. Red: P20. Orange: P27. Error bars: ± SEM. **(D)** Plot of the ratios of the width of activation in L2/3 to L4 at different TFS. Blue: P14. Red: P20. Orange: P27. Error bars: ± SEM.

Besides being propagated in the feedforward direction, stimuli elicited in L4 evoked voltage signals that were also propagated horizontally within each layer. To address whether the horizontal spread of activation is modulated by development, we analyzed line scans through L4 (Figure 4A) and L2/3 (Figure 4B) at TFS 5, 10, 20, and 30 ms. Each line scan was fit with a Gaussian curve to assess the width of signal spread within each layer (Figure 4C). In L4, the maximum width of activation was not significantly different between age groups (P14: 510 ± 117 μm; P20: 470 ± 99 μm; P27: 520 ± 118 μm; ANOVA on ranks: *p* = 0.88). Within L2/3, the maximal spread of activation was also not significantly different between groups (P14: 729 ± 133 μm; P20: 785 ± 198 μm; P27: 678 ± 108 μm; ANOVA on ranks *p* = 0.99). We conservatively chose not to analyze the horizontal spread of activation within L5 because the stimulating electrode obscured a portion of L5 from the camera. For all age groups, horizontal propagation of voltage signals within each layer remained constant, and the ratio of the lateral spread of activation in L2/3 to L4 was greater than 1 by 20 ms following stimulation, suggesting that the recurrent circuit in L2/3 is activated more broadly than that in L4 throughout the developmental window examined (Figure 4D).

### NMDA receptors account for signal duration

Excitatory synaptic transmission is significantly changed during development (Carmignoto and Vicini, 1992; Nase et al., 1999; Corlew et al., 2007; Yashiro and Philpot, 2008; Wang et al., 2012) in a way that may affect the spatio-temporal propagation of the VSD signal. To dissect the contribution of synaptic receptors to the VSD signal, we sequentially applied the NMDA receptor antagonist APV, the AMPA receptor antagonist DNQX, and the GABA_A_ receptor antagonist picrotoxin (Wang et al., 2011). Receptor-mediated components of the VSD signal were calculated by subtracting the signal recorded after application of a specific antagonist from the signal recorded prior to application of that antagonist.

**Figure 5:**
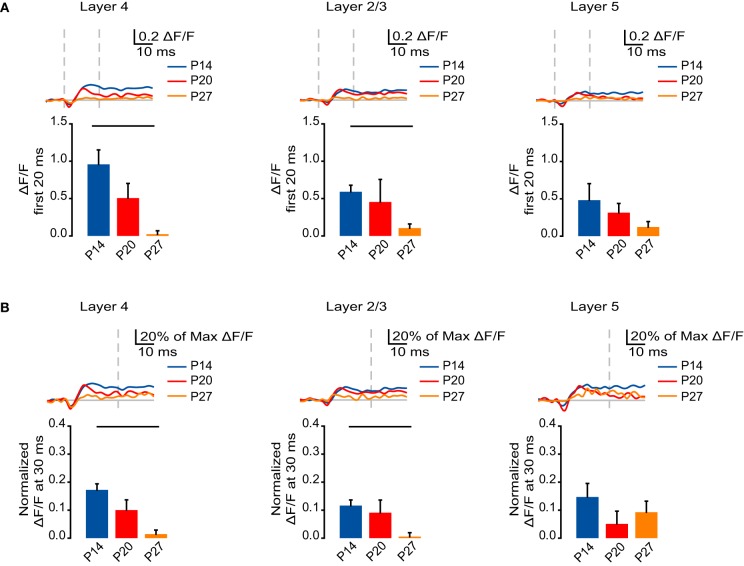
**Developmental decrease in the fast and slow NMDA receptor components of circuit activation. (A)** Top: Time course of the NMDA receptor component of the optical signals measured from ROIs in L4, L2/3, and L5 from 10 ms before stimulation to 50 ms after stimulation. The NMDA receptor-mediated component of the signal was obtained by subtracting the VSD signal remaining after perfusion of APV from the one recorded in ACSF. Blue: P14. Red: P20. Orange: P27. Light gray line: 0.0 ΔF/F. Light gray dashes: TFS 0 ms and 20 ms. Bottom: Total ΔF/F of the NMDA receptor component of the optical signal for 20 ms from stimulation measured from ROIs in L4, L2/3, and L5. Blue: P14. Red: P20. Orange: P27. Error bars: ± SEM. Dark bars indicate significant changes, *p* < 0.05. **(B)** Top: Time course of the NMDA component of the optical signals from ROIs in L4, L2/3, and L5 from 10 ms before stimulation to 50 ms after stimulation normalized to the peak ΔF/F measured in each ROI before application of synaptic blockers. Blue: P14. Red: P20. Orange: P27. Light gray line: 0.0 ΔF/F. Light gray dash: TFS 30 ms. Bottom: ΔF/F of the NMDA receptor component of the optical signal at 30 ms normalized to the peak ΔF/F before application of synaptic blockers measured from ROIs in L4, L2/3, and L5. Blue: P14. Red: P20. Orange: P27. Error bars: ± SEM. Dark bars indicate significant changes, *p* < 0.05.

NMDA receptors can mediate the spatio-temporal spread of cortical circuit activation (Laaris et al., 2000; Petersen and Sakmann, 2001). Their expression and subunit composition is developmentally regulated and confers NMDA-mediated responses with different properties (Carmignoto and Vicini, 1992; Nase et al., 1999; Corlew et al., 2007; Yashiro and Philpot, 2008). When isolated pharmacologically, the NMDA component of the VSD signal showed fast and slow components, with the fast component representing the peak activation followed by a slow component of signal persistence. We quantified the fast component of NMDA receptor-mediated activation as the sum of the ΔF/F measured at each time point during the first 20 ms following stimulation. There was a decrease in NMDA receptor-mediated component of the VSD signal in L4 across age groups, which was significant between P14 and P27 (Figure 5A; P14: 0.96 ± 0.19; P20: 0.51 ± 0.20; P27: 0.023 ± 0.046; Dunn's test: P14 vs. P27, *p* < 0.05). Similarly, in L2/3 the NMDA receptor-mediated component of the VSD signal was significantly smaller at P27 compared to P14 (Figure 5A; P14: 0.59 ± 0.087; P20: 0.45 ± 0.30; P27: 0.10 ± 0.056; Dunn's test: P14 vs. P27, *p* < 0.05). In L5, there was no significant change in the NMDA receptor-mediated component of the VSD signal across development (Figure 5A; P14: 0.48 ± 0.22; P20: 0.31 ± 0.13; P27: 0.12 ± 0.074; ANOVA on ranks: *p* = 0.21).

We next asked whether changes in the NMDA receptor-mediated signaling across development could account for changes in the persistence of the slow component of the VSD signal. The NMDA receptor component was normalized to the peak signal before application of synaptic blockers. We compared the normalized signal that remained at 30 ms, after the fast phase of the NMDA receptor-mediated signal had subsided. In both L4 and L2/3, there was a significant reduction in the persistence of NMDA receptor-mediated activation from P14 to P27 (Figure 5B; L4: P14: 0.17 ± 0.022; P20: 0.10 ± 0.037; P27: 0.014 ± 0.014; Dunn's test: P14 vs. P27, *p* < 0.05; L2/3: P14: 0.12 ± 0.020; P20: 0.091 ± 0.045; P27: 0.0055 ± 0.014; Dunn's test: P14 vs. P27, *p* < 0.05). In L5, there was no significant difference in the persistence of the NMDA receptor-mediated signal between age groups (Figure 5B; P14: 0.15 ± 0.048; P20: 0.051 ± 0.045; P27: 0.093 ± 0.039; ANOVA on ranks *p* = 0.38). These results suggest that the reduction in VSD signal persistence in L4 and L2/3 across age groups is likely related to the developmentally regulated decrease in the NMDA receptor-mediated portion of the VSD signal.

### AMPA receptor-mediated activation decreases across development

**Figure 6:**
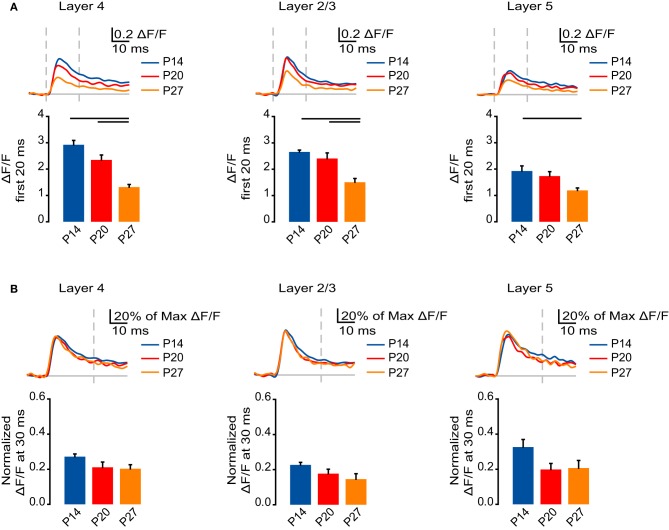
**Developmental decrease in the fast AMPA receptor component of circuit activation. (A)** Top: Time course of the AMPA receptor component of the optical signals measured from ROIs in L4, L2/3, and L5 from 10 ms before stimulation to 50 ms after stimulation. The AMPA receptor component of the signal was obtained by subtracting the VSD signal remaining after DNQX from the signal measured in ACSF with APV. Blue: P14. Red: P20. Orange: P27. Light gray line: 0.0 ΔF/F. Light gray dashes: TFS 0 ms and 20 ms. Bottom: Total ΔF/F of the AMPA receptor component of the optical signal for 20 ms from stimulation measured from ROIs in L4, L2/3, and L5. Blue: P14. Red: P20. Orange: P27. Error bars: ± SEM. Dark bars indicate significant changes, *p* < 0.05. **(B)** Top: Time course of the AMPA component of the optical signals from ROIs in L4, L2/3, and L5 from 10 ms before stimulation to 50 ms after stimulation normalized to the peak ΔF/F measured in each ROI before application of synaptic blockers. Blue: P14. Red: P20. Orange: P27. Light gray line: 0.0 ΔF/F. Light gray dash: TFS 30 ms. Bottom: ΔF/F of the AMPA receptor component of the optical signal at 30 ms normalized to the peak ΔF/F before application of synaptic blockers measured from ROIs in L4, L2/3, and L5. Blue: P14. Red: P20. Orange: P27. Error bars: ± SEM.

After blocking NMDA receptor-mediated transmission, we measured the AMPA receptor-mediated component of the VSD signal. Similar to the NMDA receptor-mediated signal, the time course of the AMPA receptor-mediated signal also had fast and slow components; we quantified the fast AMPA receptor-mediated component of the VSD signal as the sum of the ΔF/F measured at each time point during the first 20 ms after stimulation. The AMPA receptor-mediated component of the optical signal in L4 decreased significantly from P14 and P20 to P27 (Figure 6A; P14: 2.92 ± 0.17; P20: 2.35 ± 0.19; P27: 1.32 ± 0.10; Dunn's test: P14 vs. P27, *p* < 0.05; P20 vs. P27, *p* < 0.05). In L2/3, the AMPA receptor-mediated component of the signal was significantly smaller at P27 compared to P14 and P20 (Figure 6A; P14: 2.66 ± 0.073; P20: 2.41 ± 0.21; P27: 1.51 ± 0.14; Dunn's test: P14 vs. P27, *p* < 0.05; P20 vs. P27, *p* < 0.05). In L5, there was a significant reduction of the AMPA receptor-mediated VSD signal from P14 to P27 (Figure 6A; P14: 1.93 ± 0.20; P20: 1.74 ± 0.17; P27: 1.19 ± 0.095; Dunn's test: P14 vs. P27, *p* < 0.05). Therefore, the AMPA receptor-mediated component of the VSD signal decreases across development coincident with the decrease in the amplitude of the full signal.

To determine whether changes in AMPA receptor-mediated synaptic transmission contribute to changes in signal persistence, we normalized the AMPA receptor-mediated portion of the VSD signal to the peak signal measured before the application of drugs in each layer for each age group (Figure 6B). We compared the normalized signals that remained in each layer at 30 ms, after the fast phase of the VSD signal had subsided. In L4, L2/3, and L5 there were no significant differences between the normalized VSD signal remaining at each age (Figure 6B; L4: P14: 0.27 ± 0.015; P20: 0.21 ± 0.030; P27: 0.20 ± 0.023; ANOVA on ranks: *p* = 0.06; L2/3: P14: 0.23 ± 0.011; P20: 0.18 ± 0.025; 0.15 ± 0.031; ANOVA on ranks: *p* = 0.08; L5: P14: 0.33 ± 0.044; P20: 0.20 ± 0.034; P27: 0.21 ± 0.044; ANOVA on ranks: *p* = 0.06). These data are consistent with the interpretation that changes in AMPA receptor-mediated circuit activation contribute primarily to the changes in peak VSD signal; however, changes in the peak circuit activation mediated by AMPA receptors may influence the signal persistence by supporting the depolarization that leads to NMDA receptors activation.

### GABA_A_ receptor-mediated inhibition increases in layer 4 across development

The maturation of GABAergic inhibition is critical for the functional development of visual cortex (Huang et al., 1999; Li et al., 2012) and has been shown to regulate the spatio-temporal propagation of voltage signals (Laaris et al., 2000; Petersen and Sakmann, 2001; Sato et al., 2008; Wang et al., 2011). To determine possible developmental changes in the contribution of GABA_A_ receptor-mediated activity to the changes in VSD signal we analyzed the portion of the optical signal remaining after additive blockade of AMPA and NMDA receptors. As the GABA_A_ receptor blocker was applied only following complete blockade of glutamatergic synaptic transmission, our experimental design allowed us to isolate the inhibitory component of the VSD signal due to direct stimulation of local GABA releasing neurons and axons in the vicinity of the stimulating electrode (Wang et al., 2011). We quantified the GABA_A_ receptor-mediated signal as the sum of the ΔF/F measured at each time point during the first 10 ms following stimulation.

**Figure 7:**
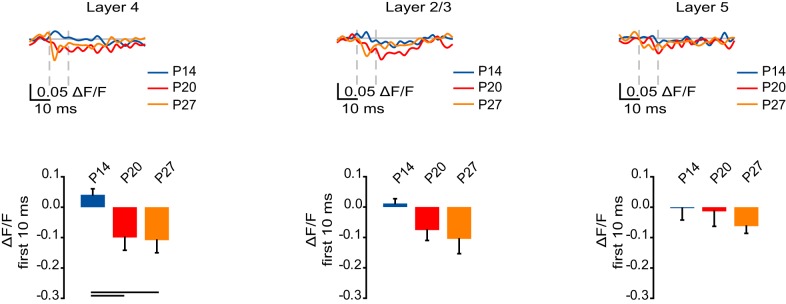
**Developmental increase in the GABA_A_ receptor component of circuit activation. Top:** Time course of the GABA_A_ receptor component of the optical signals measured from ROIs in L4, L2/3, and L5 from 10 ms before stimulation to 50 ms after stimulation. The GABA_A_ receptor-mediated component of the signal was obtained by subtracting the VSD signal remaining after perfusion of picrotoxin from the one recorded in ACSF with APV and DNXQ. Blue: P14. Red: P20. Orange: P27. Light gray line: 0.0 ΔF/F. Light gray dashes: TFS 0 ms and 10 ms. **Bottom:** Total ΔF/F of the GABA_A_ receptor component of the optical signal for 10 ms from stimulation measured from ROIs in L4, L2/3, and L5. Blue: P14. Red: P20. Orange: P27. Error bars: ± SEM. Dark bars indicate significant changes, *p* < 0.05.

In L4, the GABA_A_ receptor-mediated component of the VSD signal increased in magnitude from P14 to P20 and P27 (Figure 7; P14: 0.041 ± 0.020; P20: −0.10 ± 0.042; P27: −0.11 ± 0.041; Dunn's test: P14 vs. P20, *p* < 0.05; P14 vs. P27, *p* < 0.05). There was no significant change in the GABA_A_ receptor-mediated component of the VSD signal in L2/3 and L5, away from the site of stimulation (Figure 7; L2/3: P14: 0.012 ± 0.016; P20: −0.076 ± 0.034; P27: −0.11 ± 0.049; ANOVA on ranks: *p* = 0.06; L5: P14: −0.0029 ± 0.039; P20: −0.014 ± 0.049; P27: −0.063 ± 0.024; ANOVA on ranks: *p* = 0.27). Our data show that direct stimulation of L4 activates a larger GABA_A_ receptor-mediated VSD signal within L4 during the peak of the critical period than just after eye opening.

## Discussion

We have shown that during postnatal development there are significant changes in the spatio-temporal activation of the visual cortical circuit. Our data demonstrate that from eye opening through the fourth postnatal week, stimuli of comparable amplitude elicit smaller signals that decay more rapidly and are propagated with greater gain to other cortical layers. While the interlaminar dynamics of activation are altered, the width of activation remains unchanged. Changes in the synaptic components of the VSD signals correlate well with the modulation of spatio-temporal patterns of circuit activation.

At all ages, the sequence of circuit activation following L4 stimulation is similar. The optical signal is initially confined almost entirely to L4, before spreading primarily to L2/3 and also to L5. Upon reaching L2/3, the voltage signal spreads laterally beyond the borders of the region activated in L4. While field stimulation in L4 could depolarize axons from other layers, we did not observe robust activation outside of L4 until 5 ms after stimulation. Therefore, the majority of the optical signal in our preparation is likely due to direct stimulation of L4 and recurrent circuit activity secondary to this initial stimulation. In rat V1, L4 pyramidal neurons send axons vertically, primarily to L2/3, but also to L5 and L6, while L2/3 axons project horizontally (Burkhalter, 1989). Our optical signal follows the architectural organization of the V1 circuit.

Neural circuits must consume energy in order to propagate signals and circuits that require more voltage changes consume more energy (Ames, 2000). In an efficient neural circuit, activity is thought to become spatio-temporally constrained to maximize the ratio of information to total activation (Laughlin and Sejnowski, 2003). We show that over the course of development, the overall responsiveness to electrical stimulation of L4 decreases: we observed a reduction of both fast and persistent circuit activation. The decrease in overall activation of the circuit is partly due to an increase in GABA_A_ receptor-mediated inhibition in L4 and a decrease in AMPA and NMDA receptor-mediated excitation. Similarly, developmental increases in paired pulse depression between white matter stimulation and L2/3 depend on increasing intracortical inhibition (Rozas et al., 2001). Together, these results are consistent with synaptic development in rodent visual cortex: from the second through fourth postnatal weeks, the number of inhibitory synapses increases substantially, increasing total cortical inhibition (Blue and Parnavelas, 1983; Morales et al., 2002; Chattopadhyaya et al., 2004) and there is an approximate halving of the number of presynaptic NMDA receptors in visual cortex, which act to increase glutamatergic neurotransmitter release (Corlew et al., 2007). Therefore, changes in GABA_A_, AMPA, and NMDA-mediated currents contribute to the reduction in the fast component of the VSD signal. While neurotransmitter gated ion channels contributed substantially to the reduction in circuit excitability, they did not account for the entire reduction is the VSD signal observed from P13 to P28. Other developmental changes, including changes in intrinsic excitability and myelination, could account for the remainder of the change in excitability (Tanaka et al., 2003; Etherington and Williams, 2011; Lazarus and Huang, 2011; Wang et al., 2012).

Following sequential application of NMDA and then AMPA receptor antagonists, we show that the decrease in the signal persistence in L2/3 and L4 from P13 to P28 is mediated predominantly by NMDA receptors, although the decrease in total circuit depolarization (e.g., through AMPA receptors) may contribute to the reduced NMDA receptor activation. The timing of this NMDA receptor dependent change in circuit activation kinetics coincides with developmental decreases in NMDA receptor decay time and deactivation time (Carmignoto and Vicini, 1992; Nase et al., 1999; Yashiro and Philpot, 2008). NMDA receptor-mediated signaling did not account for changes in L5 signal persistence; consistent with our results, APV does not differentially affect synaptic transmission between connected L5 pyramidal neurons from P11 to P29 (Etherington and Williams, 2011). Therefore, by the fourth postnatal week, V1 reaches peak activation more quickly and the voltage signal becomes less persistent. These changes suggest that the efficiency of signal propagation within the V1 circuit improves during postnatal development.

While the circuit in V1 becomes less excitable and its activation less persistent during postnatal development, there is an increase in the gain of signals leaving L4. By the end of the third postnatal week, activation of L4 results in an amplified signal reaching L2/3. This circuit refinement occurs with a similar time course as the refinement of visual cortex receptive field properties (Fagiolini et al., 1994; Huang et al., 1999; Prévost et al., 2010; Espinosa and Stryker, 2012; Li et al., 2012). Intracortical amplification has been proposed as a mechanism for the development of mature receptive fields (Chance et al., 1999; Antolík and Bednar, 2011). We speculate that an increase in the gain of interlaminar circuit activation may play a role in the maturation of receptive fields. Thus, during the maturation process occurring in normal development the visual cortical circuit becomes more efficient while simultaneously developing receptive field properties via an increase in interlaminar gain.

How does the propagation of voltage signals through visual cortex compare to those through circuits in other primary sensory cortices? In insular and barrel cortex, direct stimulation of L4 elicits distinct patters of interlaminar propagation of VSD signals (Sato et al., 2008). In P15–P22 insular cortex, stimulation at the border of the dysgranular and agranular regions in “L4” results in spread to L2/3 and L5 confined to the columnar width around the stimulation site (Sato et al., 2008). While stimulation of L4 in V1 leads to the activation of L2/3 and L5, the VSD signal was greatest in L2/3, where it spread horizontally more widely than in L4. In barrel cortex at P13–P15 and P18–P22, stimulation of L4 results in a spread of depolarization similar to what we have observed in V1; however, signal spread to L5 is minimal (Petersen and Sakmann, 2001; Sato et al., 2008). As V1 and barrel cortex both have a well-defined L4 that receives most of the thalamic input (Bolz, 1994; Miller et al., 2001), it is not surprising that they show a similar responsiveness to stimulation, which is distinct from that in the agranular/dysgranular junction in insular cortex (Sato et al., 2008) where thalamic fibers distribute diffusely (Maffei et al., 2012). The differences in spread of VSD signal to L5 between visual and barrel cortex could be due to the later maturation of visual cortex relative to barrel cortex (Cheetham and Fox, 2010) or could represent differences in signal propagation between these cortices.

Our findings indicate that from the onset of visual experience, the spatio-temporal propagation of cortical activation is refined dependent upon changes in receptor-mediated synaptic transmission. The propagation of activity in the cortical circuit is modulated not only by development, but also by changes in visual experience (Palagina et al., 2009; Wang et al., 2011) a manipulation that is known to alter synaptic transmission (Morales et al., 2002; Maffei et al., 2006; Maffei and Turrigiano, 2008). Similarly, neurodevelopmental disorders that alter synaptic transmission (Harrison and Weinberger, 2005; Paluszkiewicz et al., 2011; Werner and Coveñas, 2011; Zoghbi and Bear, 2012) may disrupt the normal development of spatio-temporal circuit activation, resulting in dysfunctional propagation of stimuli through cortical circuits.

### Conflict of interest statement

The authors declare that the research was conducted in the absence of any commercial or financial relationships that could be construed as a potential conflict of interest.
